# Molluskan Hemocyanins Activate the Classical Pathway of the Human Complement System through Natural Antibodies

**DOI:** 10.3389/fimmu.2017.00188

**Published:** 2017-02-24

**Authors:** Javier Pizarro-Bauerle, Ismael Maldonado, Eduardo Sosoniuk-Roche, Gerardo Vallejos, Mercedes N. López, Flavio Salazar-Onfray, Lorena Aguilar-Guzmán, Carolina Valck, Arturo Ferreira, María Inés Becker

**Affiliations:** ^1^Immunology of Microbial Aggression Laboratory, Immunology Program, Faculty of Medicine, ICBM, Universidad de Chile, Santiago, Chile; ^2^Faculty of Medicine, Millennium Institute on Immunology and Immunotherapy, ICBM, Universidad de Chile, Santiago, Chile; ^3^Immunology Program, Faculty of Medicine, ICBM, Universidad de Chile, Santiago, Chile; ^4^Faculty of Veterinary Medicine and Livestock Sciences, University of Chile, Santiago, Chile; ^5^Biosonda Corporation, Santiago, Chile; ^6^Fundación Ciencia y Tecnología para el Desarrollo (FUCITED), Santiago, Chile

**Keywords:** molluskan hemocyanins, complement system, natural antibodies, classical pathway, carbohydrates, adjuvants

## Abstract

Molluskan hemocyanins are enormous oxygen-carrier glycoproteins that show remarkable immunostimulatory properties when inoculated in mammals, such as the generation of high levels of antibodies, a strong cellular reaction, and generation of non-specific antitumor immune responses in some types of cancer, particularly for superficial bladder cancer. These proteins have the ability to bias the immune response toward a T_h_1 phenotype. However, despite all their current uses with beneficial clinical outcomes, a clear mechanism explaining these properties is not available. Taking into account reports of natural antibodies against the hemocyanin of the gastropod *Megathura crenulata* [keyhole limpet hemocyanin (KLH)] in humans as well as other vertebrate species, we report here for the first time, the presence, in sera from unimmunized healthy donors, of antibodies recognizing, in addition to KLH, two other hemocyanins from gastropods with documented immunomodulatory capacities: *Fisurella latimarginata* hemocyanin (FLH) and *Concholepas concholepas* hemocyanin (CCH). Through an ELISA screening, we found IgM and IgG antibodies reactive with these hemocyanins. When the capacity of these antibodies to bind deglycosylated hemocyanins was studied, no decreased interaction was detected. Moreover, in the case of FLH, deglycosylation increased antibody binding. We evaluated through an *in vitro* complement deposition assay whether these antibodies activated the classical pathway of the human complement system. The results showed that all three hemocyanins and their deglycosylated counterparts elicited this activation, mediated by C1 binding to immunoglobulins. Thus, this work contributes to the understanding on how the complement system could participate in the immunostimulatory properties of hemocyanins, through natural, complement-activating antibodies reacting with these proteins. Although a role for carbohydrates cannot be completely ruled out, in our experimental setting, glycosylation status had a limited effect. Finally, our data open possibilities for further studies leading to the design of improved hemocyanin-based research tools for diagnosis and immunotherapy.

## Introduction

Hemocyanins, present in some mollusks, are respiratory glycoproteins, found freely dissolved in the hemolymph of these organisms (1), where they are also involved in immune functions (2). Unlike hemoglobin, hemocyanins bind oxygen through copper (II) atoms, leading to their characteristic blue color (1, 3). Depending on the organism, hemocyanins can be found arranged commonly into decamers, didecamers, and tridecamers, with a “hollow cylinder” configuration, although more recently, megatridecamers, with a “filled cylinder” shape, have also been reported (4, 5).

Hemocyanins are highly glycosylated [up to 9% in weight (1)], and among the most studied is the keyhole limpet hemocyanin (KLH, from the gastropod *Megathura crenulata*), with immunostimulatory properties described around 50 years ago (1). These properties have led to its use in many applications, including experimental antigen, assessment of immunocompetence in humans, carrier for antibody generation against diverse hapten molecules and peptides, diagnostic tool for the parasite *Schistosoma mansoni*, unspecific immunostimulant, and adjuvant in various vaccine formulations (6). Of special interest is its use in superficial bladder carcinoma, where intravesical instillation delivers better results than the classical treatment with bacillus Calmette-Guérin and with less side effects (7).

However, more recently, we have described *Concholepas concholepas* hemocyanin [CCH (8)] from the *Loco* or Chilean abalone and *Fissurella latimarginata* hemocyanin [FLH (9)], from the *Lapa negra*, gastropod mollusks found along the Pacific coasts of Chile and Perú. These hemocyanins possess immunogenicity and antitumor immunostimulatory properties, equal to or better than those of KLH (10, 11). CCH has been used as a carrier for antibody generation (12–15), as an experimental antigen (16, 17); as a carrier for an anti-prion vaccine (18), an immunocontraceptive vaccine (19); and more recently, as an adjuvant in a phase I trial for a dendritic cell vaccine against prostate cancer (20) In spite of this, the molecular mechanisms behind these remarkable properties remain elusive. Some previous explanations, invoking characteristics such as elevated size or quasi-D5 symmetry, do not seem now entirely satisfactory since isolated subunits have the same effect or can even outperform the whole molecules in some aspects (21, 22). Other explanations are their xenogenicity, their increased permanence time inside antigen-presenting cells as recently proposed by us (11), and their high carbohydrate content.

Although KLH glycosylation profiles are relatively well known, studies in the matter have not provided a definitive answer with regard to their role in immunogenicity/antigenicity. Early studies showed that KLH has carbohydrate epitopes that can cross-react with tumor antigens, leading to the antitumor effect observed for KLH (23). More recent studies show that KLH induces the activation and maturation of human dendritic cells, and that these properties are partially mediated by the mannose receptor, which can be explained by the presence in KLH of mannose and fucose, ligands of this receptor (24), as well in CCH and FLH, where mannose receptor mediated their endocytosis in murine peritoneal macrophages (25). By contrast, our previous work has shown that deglycosylation of FLH decreases its antitumor activity (9), while in CCH does not affect it and it even increases its immunogenicity (11).

Natural antibodies in experimental animals (26), birds (27–29), cattle (30, 31), and humans (26, 32, 33) bind to KLH, thus explaining, at least partially, the immunomodulatory properties of hemocyanins. These antibodies may activate the complement system, with consequences in innate and adaptive arms of the immune response (34, 35). C1, a pattern recognition receptor present in the serum of vertebrates, initiates the classical pathway of the complement system by detecting antigen-bound antibodies, leading to the sequential activation of its serine-proteases C1r and C1s. The latter then cleaves C2 and C4, thus generating classical pathway C3 convertases which lead to terminal pathway activation and generation of membrane attack complexes which lyse the target cell (34, 35). During this activation, proinflammatory, opsonizing, and immunostimulatory functions are also generated, all of them highly relevant in a normal immune response (34). Together with the lectin pathway (MBL and ficolins) and the alternative pathway, this system provides a very important defense against bacterial cells, viruses, and transformed cells (35).

The presence of natural antibodies against one hemocyanin (KLH) has been known for some time now (26, 33). However, studies on possible connections between these antibodies and complement are scarce and need to be revisited under the present state of knowledge of this system. KLH has been used as a model antigen in a study of complement activation (36), but the complement-activating capacity of other hemocyanins has not been addressed. Moreover, the conditions described in a previous study do not discriminate between the classical and the lectin pathways as sources of the observed complement deposition (36).

For these reasons, we propose that natural antibodies against CCH and FLH (similar to natural anti-KLH antibodies), are detectable in healthy donors and that they specifically activate the classical pathway of the complement system. Accordingly, IgG and IgM antibodies were detected, being able to recognize all three hemocyanins mostly through non-glycosylated sites. These antibodies activated *in vitro* the classical pathway of the complement system, offering at least a partial explanation for the remarkable immunomodulatory properties of these glycoproteins.

## Materials and Methods

### Hemocyanins

Lyophilized KLH (in PBS, 0.1 M sodium phosphate, 0.15 M NaCl, pH 7.2 after reconstitution) was obtained from Thermo Scientific (Waltham, MA, USA). CCH and FLH, isolated and purified under sterile and pyrogen-free conditions, were provided by Biosonda Company (Santiago, Chile). KLH was first reconstituted in distilled water and then all hemocyanins were diluted with PBS to desired concentrations. To obtain deglycosylated hemocyanins, oxidation with periodate was performed, as reported previously by us (11). Briefly, 15 mM sodium periodate (Merck, Germany) in 0.1 M sodium acetate buffer was prepared, adjusted to pH 5.5, and used to dilute the different hemocyanins to 0.5 mg/ml. They were then incubated for 1 h, in the dark, at room temperature and finally an excess of ethylene glycol was added to consume the remaining periodate by overnight incubation. Then, the deglycosylated hemocyanins (termed Ox-KLH, Ox-CCH, and Ox-FLH) were concentrated using the Amicon Ultra-4 10K (Merck) system, its concentration determined by the Bradford method (37) and diluted in PBS to desired concentrations.

### Patient and Donor Sera

Sera from patients P80 and P202, corresponding to an adult male and an adult female, respectively, who received immunotherapy against melanoma [consisting of tumor antigen-pulsed dendritic cells TAPCells (38, 39) and KLH as an adjuvant], were used as positive controls for the presence of antibodies against KLH. Blood was also obtained from five healthy adult donors D1 to D5 (three females and two males) with no diagnosis of chronic conditions and not subjected to present or past active immunizations with molluskan hemocyanins. Decomplemented serum was obtained by heat inactivation at 56°C, for 30 min.

### Assessment of Human Antibody Binding to Molluskan Hemocyanins

Ninety-six-well polystyrene microplates were incubated with the different hemocyanins (native and deglycosylated) and with casein as a negative control, all at 10 µg/ml in PBS, through overnight incubation at 4°C. After washing with PBS, 0.05% Tween-20 (PBS-T), active solid phase sites were blocked with a 2.5% w/v casein (Winkler, Santiago, Chile) solution with a pH of 7.0, at 37°C for 2 h. Then, dilutions of 1:100 of decomplemented sera from patients and healthy donors, in blocking solution were incubated at 37°C for 1 h. Rabbit anti-human immunoglobulins (Amersham Biosciences, Buckinghamshire, UK), goat anti-human IgG (specific for Fc_γ_ chain, Sigma-Aldrich, St. Louis, MO, USA), or goat anti-human IgM (specific for Fc_5µ_ fragments, Jackson ImmunoResearch, West Grove, PA, USA), all coupled to peroxidase, were added diluted in PBS-T. The reaction was developed with ABTS and 0.1% H_2_O_2_, and read at 405 nm after 20 min of reaction.

### Assessment of Complement Classical Pathway Activation by Molluskan Hemocyanins

Microplates were sensitized as indicated above, and wells with purified human IgM (Jackson ImmunoResearch) at a concentration of 2 µg/ml, were also included, followed by washings with Tris-buffered saline (TBS) with 0.05% Tween-20 and 5 mM CaCl_2_ (TBS-T-Ca^2+^). Casein was used for blocking, and diluted sera from unimmunized donors were added. Then, the wells were incubated with purified human C1 (CompTech, Tyler, TX, USA) at 200 ng/ml in Veronal-buffered saline (VBS; 5 mM sodium diethylbarbiturate, 140 mM NaCl, 0.5 mM MgCl_2_, and 0.15 mM CaCl_2_) and then with C4 (CompTech), at 1 µg/ml, also in VBS. Then, a goat anti-human C4 antibody (CompTech) was added diluted in 2.5% casein, followed by a rabbit anti-goat IgG coupled to peroxidase (Calbiochem, Billerica, MA, USA), diluted in TBS-T-Ca^2+^. Finally, the reaction was developed with ABTS and 0.1% H_2_O_2_ and read at 405 nm after 5 min of reaction. EDTA was used in some experiments at a 20 mM concentration and an anti-C1q antibody [anti-C1q-85 (40), kindly provided by Dr. Diana Wouters, Immunopathology Department, Sanquin Blood Supply Foundation, Amsterdam, The Netherlands] was used at 2 µg/ml.

### Ethics Statement

This study was carried out in accordance with the recommendations of the National Commission for Science and Technology (CONICYT) guidelines. All subjects gave written informed consent in accordance with the Declaration of Helsinki. The protocol was approved by the Bioethical Committee for Human Research of the University of Chile.

### Statistical Analysis

GraphPad Prism 5 (GraphPad Software, San Diego, CA, USA) was used to process and analyze data and results. Statistical analysis was performed by means of a two-tailed Student’s *t*-test for both antibody detection and classical pathway activation assays. All experiments were replicated at least three times with representative results shown.

## Results

### Antibodies That Recognize Diverse Molluskan Hemocyanins Are Present in Human Healthy Donors

**Figure 1:**
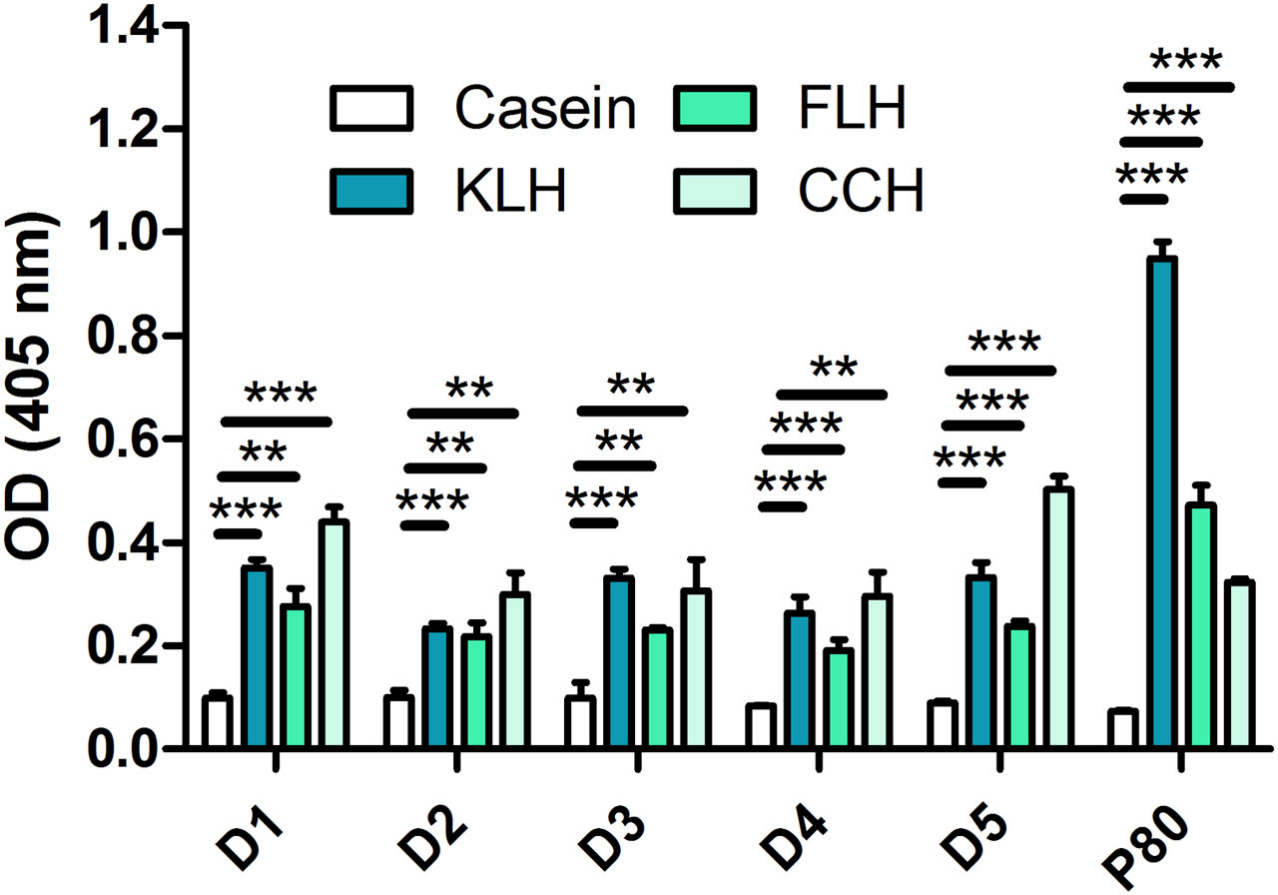
**Unimmunized donors present natural antibodies against molluskan hemocyanins**. The levels of immunoglobulins specifically binding to keyhole limpet hemocyanin (KLH), *Fissurella latimarginata* hemocyanin (FLH), and *Concholepas concholepas* hemocyanin (CCH) were determined by an indirect ELISA in sera from five unimmunized donors, at a dilution of 1:100. Serum from a patient immunized with KLH (P80) was used as positive control. Bars represent the mean of three wells with the respective SD. The figures in this work are representative of at least three independent experiments. **p ≤ 0.01; ***p ≤ 0.001, assessed by a two-tailed Student’s *t*-test.

**Figure 2:**
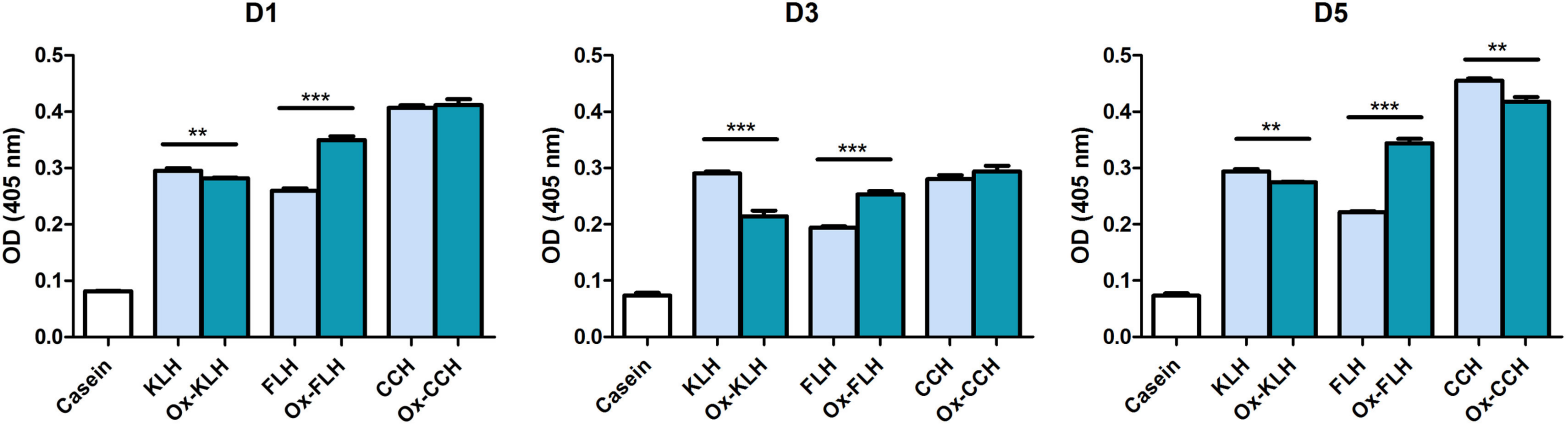
**Unimmunized donors present natural antibodies against native and deglycosylated hemocyanins**. The levels of immunoglobulins binding to keyhole limpet hemocyanin (KLH), *Fissurella latimarginata* hemocyanin (FLH), and *Concholepas concholepas* hemocyanin (CCH) and their deglycosylated counterparts [deglycosylated (oxidized) KLH (Ox-KLH), deglycosylated (oxidized) FLH (Ox-FLH), and deglycosylated (oxidized) CCH (Ox-CCH), respectively] were determined by an indirect ELISA in sera from unimmunized donors D1, D3, and D5. **p ≤ 0.01; ***p ≤ 0.001, assessed by a two-tailed Student’s *t*-test.

According to previous reports (26, 33), we found antibodies recognizing KLH in the five unimmunized donors. We also found antibodies (total immunoglobulins) that recognized hemocyanins with recently described biomedical potential, such as FLH and CCH (Figure 1). This is the first report on natural antibodies against molluskan hemocyanins other than KLH. The positive anti-KLH control sera (P80), obtained from a melanoma patient immunized with tumor antigen-pulsed dendritic cells TAPCells (38, 39) and KLH as an adjuvant, also reacted with CCH and KLH. Then, we performed the same assays for donors D1, D3, and D5 (since they consistently showed higher reactivity) but using deglycosylated hemocyanins, since it is accepted that natural antibodies usually present broad specificity for carbohydrates. Interestingly, although deglycosylation sometimes decreased antibody binding, especially for KLH in donor D3, in some cases, deglycosylation markedly increased antibody binding, especially in deglycosylated (oxidized) FLH (Ox-FLH) (Figure 2).

**Figure 3:**
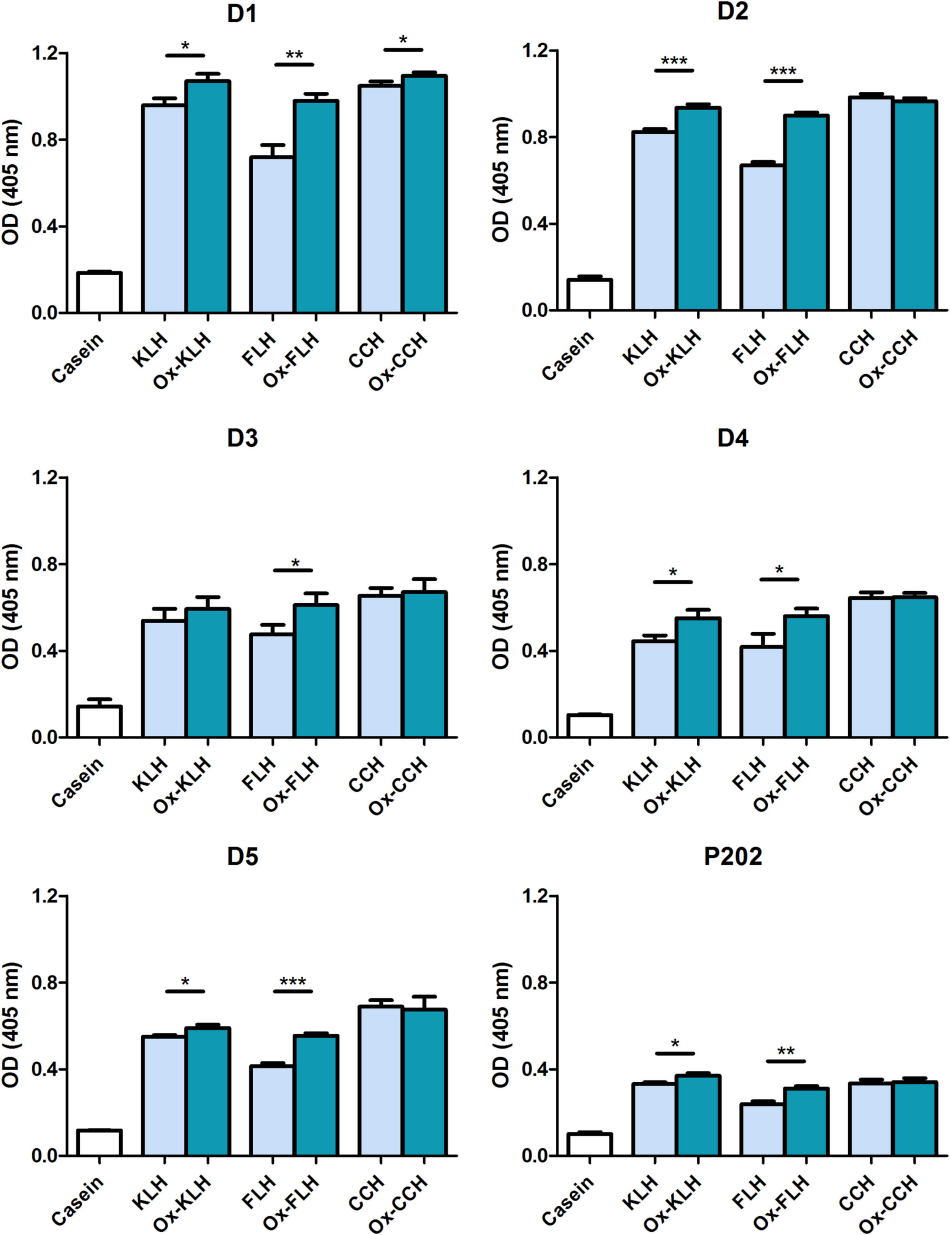
**Unimmunized donors present natural IgM antibodies against native and deglycosylated hemocyanins**. The levels of IgM immunoglobulins binding to keyhole limpet hemocyanin (KLH), *Fissurella latimarginata* hemocyanin (FLH), and *Concholepas concholepas* hemocyanin (CCH) and their deglycosylated counterparts [deglycosylated (oxidized) KLH (Ox-KLH), deglycosylated (oxidized) FLH (Ox-FLH), and deglycosylated (oxidized) CCH (Ox-CCH)] were determinated by an indirect ELISA in sera from five donors, using a specific anti-Fc_5µ_ antibody. Serum from P202 was used as a positive control at a dilution of 1:250. *p ≤ 0.05; **p ≤ 0.01; ***p ≤ 0.001, assessed by a two-tailed Student’s *t*-test.

In order to assess the nature of these antibodies, we studied the binding of IgM class antibodies, classically described as the most common natural antibodies (41), using a specific antibody against IgM. Figure 3 shows that all donors presented IgM antibodies against native and deglycosylated hemocyanins. As previously observed, deglycosylation did not decrease antibody binding in any case, leading to a consistent increase in binding to Ox-FLH and to a lesser degree to deglycosylated (oxidized) KLH (Ox-KLH).

**Figure 4:**
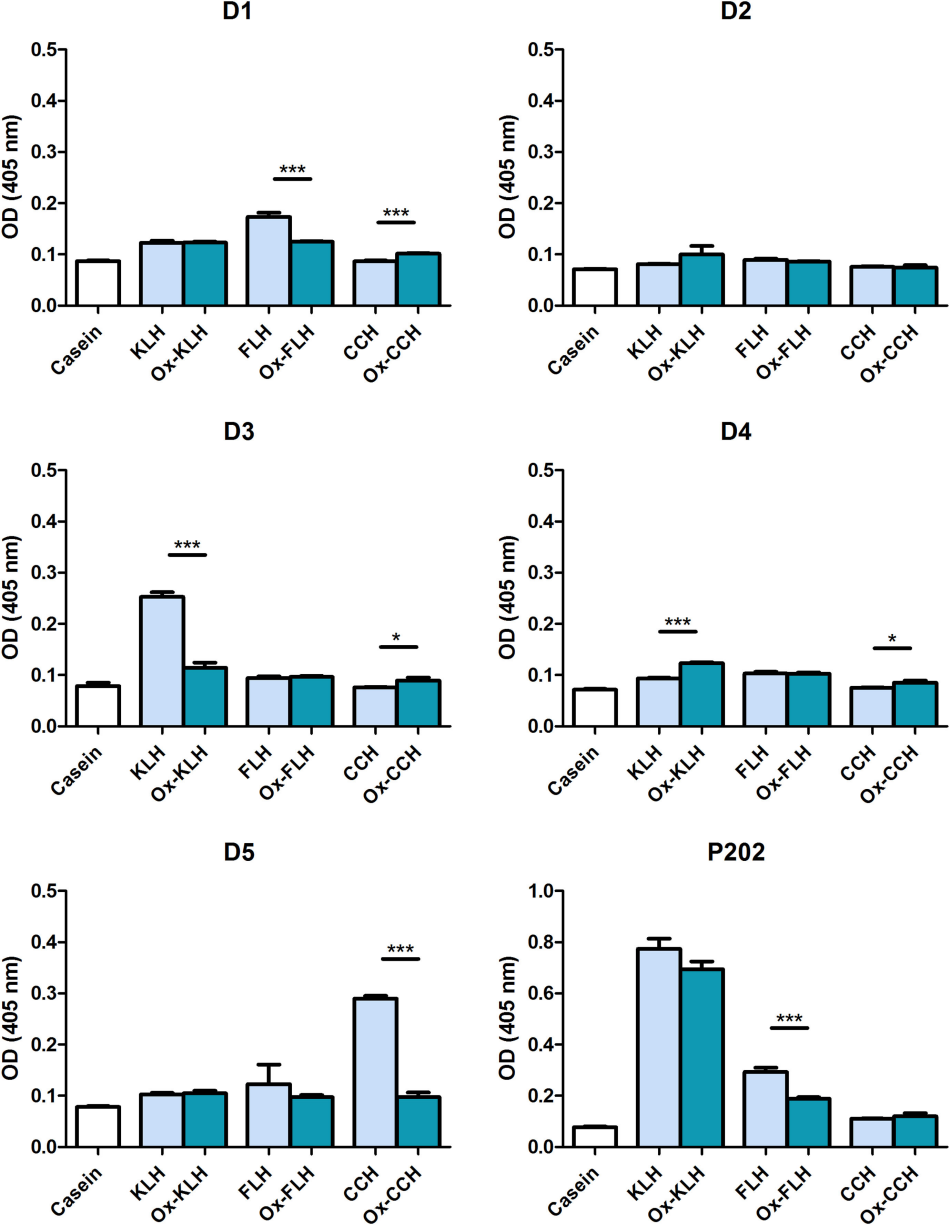
**Unimmunized donors present natural IgG antibodies against native and deglycosylated hemocyanins**. The levels of IgG immunoglobulins binding to keyhole limpet hemocyanin (KLH), *Fissurella latimarginata* hemocyanin (FLH) and *Concholepas concholepas* hemocyanin (CCH) and their deglycosylated counterparts [deglycosylated (oxidized) KLH (Ox-KLH), deglycosylated (oxidized) FLH (Ox-FLH), and deglycosylated (oxidized) CCH (Ox-CCH)] were determinated by an indirect ELISA in sera from five donors, using a specific anti-Fc_γ_ antibody. Serum from P202 was used as a positive control at a dilution of 1:250. *p ≤ 0.05; ***p ≤ 0.001, assessed by a two-tailed Student’s *t*-test.

We then studied specific IgG binding to native and deglycosylated hemocyanins. Figure 4 shows that two donors (D3 and D5) showed the highest IgG reactivity against KLH and CCH, respectively, although lower than those shown for IgM. Differently from IgM, deglycosylation strongly decreased IgG binding to control levels in D3 and D5. D5 was the only donor who presented a change on total immunoglobulin levels against CCH after deglycosylation, and this was not associated to a change in IgM levels, but to the mentioned decrease in IgG levels.

### Molluskan Hemocyanins Activate the Classical Pathway of the Human Complement System through Antibodies Present in Healthy Donors

**Figure 5:**
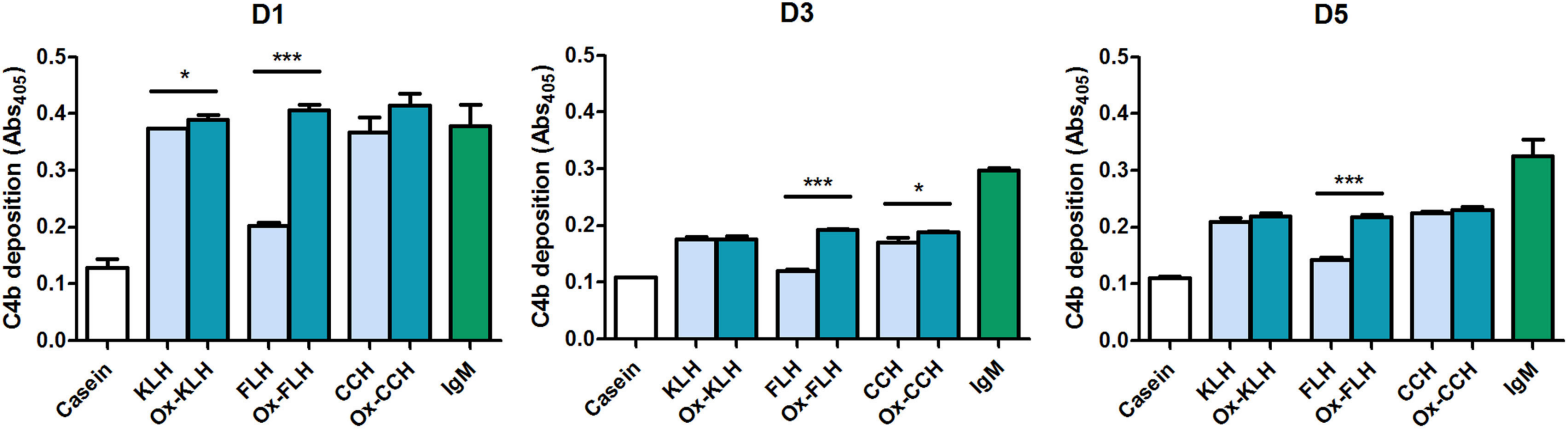
**Sera from unimmunized donors interact with hemocyanins and activate *in vitro* the classical pathway of the human complement system**. A C4b deposition assay was used to determine the activation of the classical pathway when keyhole limpet hemocyanin (KLH), *Fissurella latimarginata* hemocyanin (FLH), *Concholepas concholepas* hemocyanin (CCH), and their deglycosylated counterparts [deglycosylated (oxidized) KLH (Ox-KLH), deglycosylated (oxidized) FLH (Ox-FLH), and deglycosylated (oxidized) CCH (Ox-CCH)] were incubated with sera from three unimmunized donors, at a dilution of 1:60 and human purified IgM was used as a positive control. *p ≤ 0.05; ***p ≤ 0.001, assessed by a two-tailed Student’s *t*-test.

We then used a C4b deposition assay to assess classical pathway activation by molluskan hemocyanins. We used serum from donors D1, D3, and D5 as a source of antibodies, since these donors presented both IgM and IgG antibodies. As seen in Figure 5, the three hemocyanins (both native and deglycosylated) activated the classical pathway of the human complement system, although a marked difference can be observed for native and deglycosylated FLH in all three donors. No activation was observed in the absence of patient serum or C1 (data not shown). No decrease in C4b deposition was observed upon CCH deglycosylation, an indication that the carbohydrate-binding IgG antibodies observed for D3 and D5 in Figure 4 do not activate the complement system.

**Figure 6:**
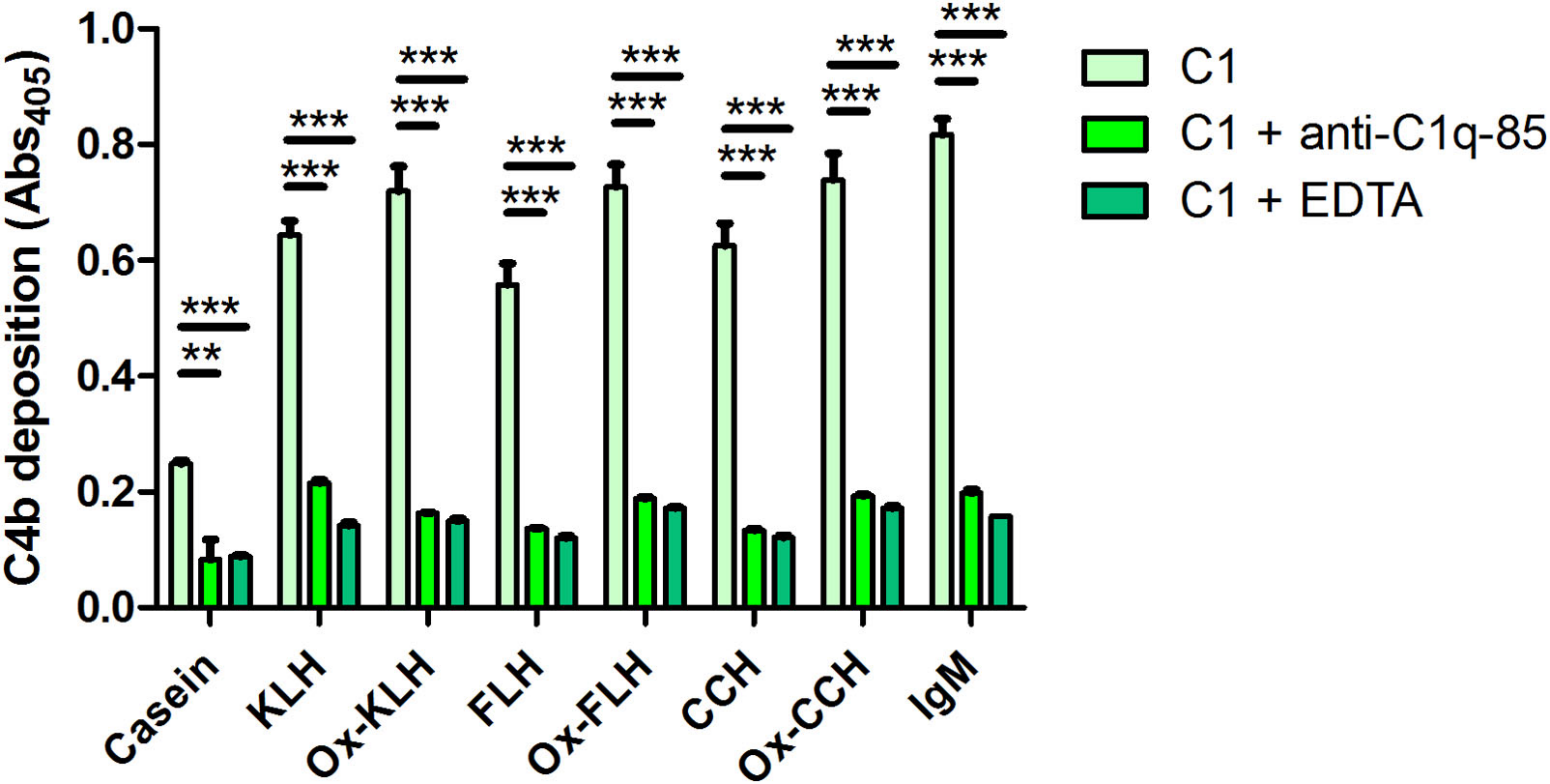
**The activation of the classical pathway of the human complement by hemocyanins depends on C1 binding to immunoglobulins**. A C4b deposition assay was used to determine the activation of the classical pathway when keyhole limpet hemocyanin (KLH), *Fissurella latimarginata* hemocyanin (FLH), *Concholepas concholepas* hemocyanin (CCH), and their deglycosylated counterparts [deglycosylated (oxidized) KLH (Ox-KLH), deglycosylated (oxidized) FLH (Ox-FLH), and deglycosylated (oxidized) CCH (Ox-CCH)] were incubated with sera from D1, in conditions similar to those used in the previous experiments, or in the presence of EDTA (classical and lectin pathway inhibitor) or anti-C1q-85 (an antibody that prevents C1q binding to Fc regions of immunoglobulins). **p ≤ 0.01; ***p ≤ 0.001, assessed by a two-tailed Student’s *t*-test.

Finally, to rule out unspecific interactions leading to C4b deposition, with the same C4b deposition assay, we used an anti-C1q-85 antibody, directed against the globular portions of C1q, thus preventing C1q binding to immunoglobulins and consequent C1 activation (40). Due to a limited availability of anti-C1q-85, this assay was performed with serum from D1, which consistently showed higher levels of C4b deposition. As shown on Figure 6, anti-C1q-85, even at the relatively low concentration of 2 µg/ml, decreased C4b deposition to levels similar to negative EDTA controls. Thus, as expected, the classical pathway activation described for hemocyanins is dependent on the C1 binding to immunoglobulins, further discarding alternative or unknown mechanisms, at least in these settings.

## Discussion

In spite of the numerous biomedical and biotechnological applications for hemocyanins, especially for KLH (1, 2), molecular information to explain the potent immunomodulatory properties of these proteins is scarce. In this study we tested whether (i) natural antibodies described for KLH (26, 33) are also present against CCH and FLH, with emerging biomedical relevance and (ii) binding of these antibodies activates the classical pathway of the human complement system, as a possible hemocyanin-mediated immunomodulatory mechanism. We show here the presence of anti-KLH, anti-CCH, and anti-FLH natural antibodies in unimmunized, healthy donors, and that these antibodies activate the classical pathway of the complement system.

The results on Figure 1 agree with those obtained for KLH in other species (26, 27, 30) and we also detected FLH- and CCH-binding antibodies in all donors. These results are explained, at least partly, by common structural characteristics of these hemocyanins, such as shared preserved xenogeneic peptide sequences and the presence of mannose-rich structures, among other carbohydrates (6). These results announce the possibility that, in a large scale study, similar results may prevail. It would also be relevant to evaluate whether different levels of these antibodies, as seen in other species (29, 31), correlate with responses against pathogenic challenges or with differential responses against these hemocyanins. These evaluations should be experimentally performed (i.e., against CCH, KLH, or FLH used as carrier proteins for antibody generation), and in therapeutic contexts, such as CCH, used as an adjuvant in antitumor dendritic cell vaccines or KLH in melanoma (42).

Natural antibodies bind a wide range of carbohydrates (43–45), a situation similar to that observed for natural anti-KLH antibodies (46). However, when we performed the same assay with deglycosylated hemocyanins (Figure 2), only decreases in binding to KLH (especially in D3) and CCH were observed. Furthermore, differently from natural antibodies, FLH deglycosylation increased antibody binding in most donors, as reported by us for CCH in mice (47). Widely used, periodate-mediated deglycosylation is supposed to remove all types of carbohydrates. However, another glycosylated protein (48), subjected to periodate oxidation, still binds to specific lectins that recognize relevant carbohydrate epitopes. This indicates that the periodate oxidation could be actually unmasking carbohydrate epitopes, preventing the decrease of antibody binding and sometimes effectively increasing this type of binding. It is also feasible that due to a higher microheterogeneity of N-linked sugars compared to KLH, and possibly residues of sialic acid, FLH presents a differential susceptibility to periodate-mediated deglycosylation. Future studies are in part aimed to elucidate this deglycosylation-induced antibody binding.

Natural antibodies usually belong to the IgM class and we detected this immunoglobulin class in all donors, recognizing both native and deglycosylated hemocyanins (Figure 3). However, similar to the previous experiment, deglycosylation led to an increase in binding to FLH and KLH. It is also relevant the fact that the presence of IgG natural antibodies recognizing KLH has also been described (26). Figure 4 shows that the sera from two donors (D3 and D5) recognize some hemocyanins. Differently from what occurs with IgM, in both cases, deglycosylation does lead to a sharp decrease in reactivity, an indication that IgG antibodies in these donors are indeed directed against carbohydrates. However, their physiological relevance remains to be assessed since their levels seem to be lower as compared to IgM.

The levels of all these antibodies are relatively modest, as usually described for natural antibodies. As a comparison, P80, the positive control, a patient immunized with KLH in the context of a dendritic cell vaccine, has a much higher reactivity, even at the dilutions shown (1:100 in donors vs. 1:200 in P80). However, even at these low levels, natural antibodies can have a profound impact in early responses against pathogens (41). It also seems relevant that the reactivity observed for P202 does not seem to be mediated by natural antibodies, since its IgG reactivity is much higher than those detected in sera from donors D3 and D5 (Figure 4). This is expected in an actively immunized patient.

Complement activation is a fundamental arm in immune responses, as both a danger signal detector and as an amplification mechanism (34, 35). It has been reported that natural antibodies in human serum bind KLH, leading to C3 deposition (36). However, this report does not discriminate between the different activation pathways, although the alternative pathway is not efficiently activated by fluid phase molecules. This led us to perform experiments assessing the exclusive contribution of the classical pathway through a complement deposition assay with a reconstituted system, and with three different hemocyanins. Figure 5 shows that all hemocyanins activate the complement system in sera from all donors, and this activation is mediated by C1 binding to immunoglobulins (as seen in Figure 6), as classically described (35). For KLH and CCH, deglycosylation only led to minor changes in complement deposition for D1 and D3 donors, respectively. However, FLH deglycosylation, in agreement with the antibody-binding experiments, led to increased classical pathway-mediated complement activation in the three donors, indicating that at least some of the antibodies detected generate C1-binding sites. For D5 donor, even though IgG antibody binding diminishes upon deglycosylation, the latter does not affect complement deposition, indicating that these antibodies may belong to IgG subclasses that do not activate the complement system, such as IgG2 or IgG4.

Considering that natural antibodies activated the classical pathway of the complement system in the presence of hemocyanins, this adds a new potential explanation for the immunostimulatory properties of these proteins. Of course, more experiments, especially *in vivo*, are required to establish this as a physiologically relevant phenomenon. Future studies in our laboratories will address the possibility that the lectin pathways are activated by molluskan hemocyanins, since it is feasible that MBL and ficolins may bind to sugars present on these proteins.

Deglycosylated (oxidized) FLH actually shows increased antibody binding, and concomitantly increased complement deposition. Therefore, Ox-FLH could be considered a valid, improved alternative for uses mediated by antibody binding and complement activation (e.g., as a carrier for monoclonal antibody generation). In addition, FLH, unlike KLH and CCH, is constituted by only one subunit (9), a convenience in a biotechnological product, in terms of uniformity and amenability for GMP production.

In synthesis, this work proposes a potential mechanism that could contribute to the immunomodulatory properties of molluskan hemocyanins, by describing natural, complement-activating antibodies reacting with hemocyanins such as CCH and FLH, with emerging biomedical relevance. This information opens a number of possibilities for further studies that may prove useful in the design of better hemocyanin-based research tools, for diagnosis and immunotherapy.

## Author Contributions

Conception and design: JP-B, CV, AF, and MB. Performed the experiments and data acquisition: JP-B, LA-G, ES-R, and GV. Data analysis and interpretation: JP-B, IM, LA-G, ES-R, GV, CV, AF, and MB. Production of critical reactives: IM, ML, FS-O, MB, and AF. Manuscript writing: JP-B, AF, and MB. All the authors read and approved the final version of this manuscript.

## Conflict of Interest Statement

The authors declare that the research was conducted in the absence of any commercial or financial relationships that could be construed as a potential conflict of interest.

## Abbreviations

KLH, keyhole limpet hemocyanin; Ox-KLH, deglycosylated (oxidized) KLH; FLH, *Fissurella latimarginata* hemocyanin; Ox-FLH, deglycosylated (oxidized) FLH; CCH, *Concholepas concholepas* hemocyanin; Ox-CCH, deglycosylated (oxidized) CCH.
